# Injuries Following Segway Personal Transporter Accidents: Case Report and Review of the Literature

**DOI:** 10.5811/westjem.2015.7.26549

**Published:** 2015-10-20

**Authors:** John Ashurst, Benjamin Wagner

**Affiliations:** Conemaugh Memorial Medical Center, Department of Emergency Medicine, Johnstown, Pennsylvania

## Abstract

The Segway® self-balancing personal transporter has been used as a means of transport for sightseeing tourists, military, police and emergency medical personnel. Only recently have reports been published about serious injuries that have been sustained while operating this device. This case describes a 67-year-old male who sustained an oblique fracture of the shaft of the femur while using the Segway® for transportation around his community. We also present a review of the literature.

## INTRODUCTION

In 2001, Dean Kamen developed a self-balancing, zero emissions personal transportation vehicle, known as the Segway® Personal Transporter (PT).^1^ The Segway’s® top speed is 12.5mph and was deemed safe for operation on urban pedestrian areas by the Centre for Electric Vehicle Experimentation in Quebec in 2006.^1^,^2^ However, several reports have been published that showed serious injuries to the “gliders” who operate these devices.^3^–^6^ This report adds to the growing literature of serious injury associated with the Segway® Personal Transporter.

## CASE REPORT

A 67-year-old male presented to the emergency department with right leg pain after a fall from his Segway®. The patient reported that he used the personal transporter as his main means of transportation around the community and that evening had several alcoholic drinks and attempted to drive home. En route, he subsequently fell from the Segway® and injured his right leg. Past medical history was significant for diabetes and coronary artery disease.

Physical exam revealed a temperature of 36.6, pulse of 72 beats per minute, respirations of 14 and a blood pressure of 176/94mmHg. The patient’s Glasgow coma scale was 15 and he did not appear to have an alcohol smell on his breath. The only outward signs of trauma were located on his right lower extremity. A gross deformity was noted over the mid thigh with the entire lower extremity held in flexion and external rotation. Peripheral pulses were present in the extremity and no parasthesia was noted.

Radiograph of the right femur demonstrated an oblique fracture of the proximal shaft of the femur with severe displacement and angulation (Figure). Alcohol level was 0.024% and the remainder of the trauma studies were negative. The patient was subsequently admitted to the trauma service and underwent operative fixation the next day. He was discharged to a rehab facility five days post injury.

## DISCUSSION

The U.S. Consumer Product Safety Commission is tasked in the United States with compiling data in the National Electronic Injury Surveillance System on injuries related to consumer products. Despite two separate recalls issued by the commission on the Segway® Personal Transporter, only 33 injuries were noted in the National Electronic Injury Surveillance System (NEISS) cases when searched with the key term “Segway”® from the year 2009 to 2013.^7^ Few injuries were identified because the National Electronic Injury Surveillance System does not have a specific code for this means of transportation but includes it with Scooters/Skateboards-powered under the code 5042.

When compared to published data from case reports and case series, none of the NEISS match the published literature. Most likely, the scarcity of literature is related to the under-reporting of the true number of accidents while using personal transporters. This is evidenced by the lack of an International Classification of Diseases 10 code as well as only a handful of reported cases.

After a review of Medline, we found four separate publications that noted significant injuries in relation to the Segway® Personal Transporter (Table 1). Of those reviewed, 16 patients required hospital admission due to significant traumatic injuries and seven were placed in an intensive care setting. Further examination showed that much like our patient, 81% of patients had a fracture with 38% occurring in the lower extremity. Although fractures are common, this classically differs from skateboard and scooter injuries in which the majority occur in the upper extremity.

More alarmingly, however, is the age of those sustaining injuries. Based upon reported data in the literature and from the NEISS, the average age of those injured is 46.07 years old (Table 2) on a personal transporter. Also, 44% of those reported injuries on personal transporters had significant head trauma that required an intensive care admission. It is difficult to ascertain the reason for this trend but could be related to personal transporters being used more by tourists as compared to other modes of transportation.

No deaths caused by Segway® use could be found in the published medical literature or within the NEISS over the time period selected. Ironically, however, a subsequent owner of the Segway® company perished after his personal transporter rolled off a 30-foot cliff and into the water in the United Kingdom.^8^

## CONCLUSION

Based upon a literature review, injuries from the Segway® Personal Transporter are likely under-reported but those that are reported are significant in nature. Emergency physicians and the Consumer Product Safety Commission should continue to monitor the number of injuries that present in the United States, and further studies regarding the personal transporter’s safety should be undertaken.

## Figures and Tables

**Figure:**
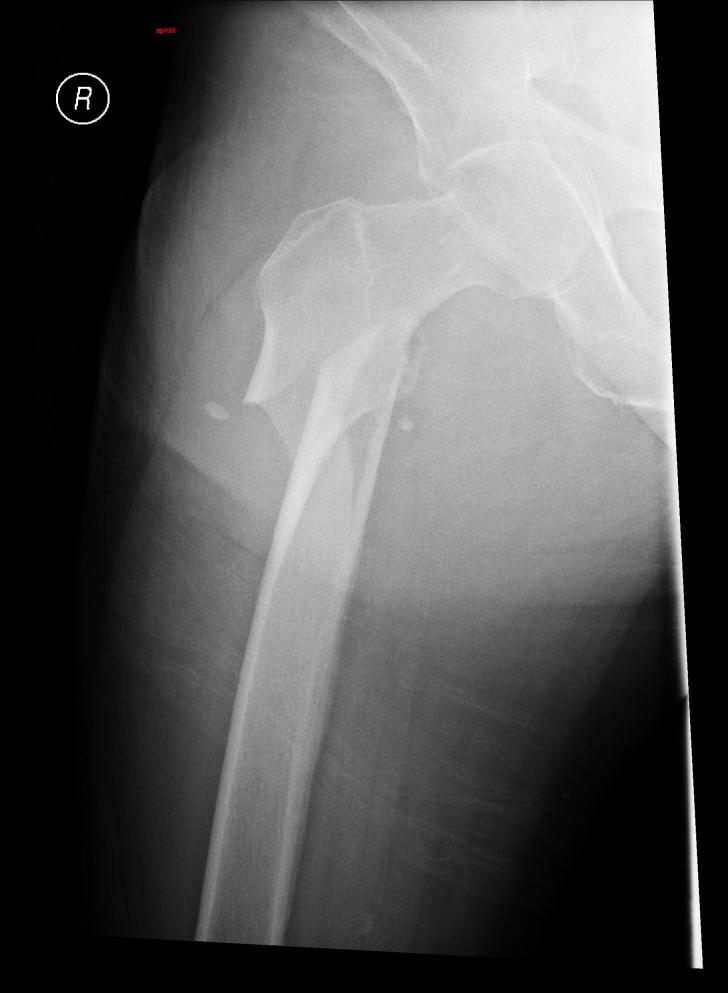
Oblique fracture of the proximal shaft of the femur with severe displacement and angulation.

**Table 1:** Compiled data from all published reports on traumatic Segway® injuries requiring admission (n=16).

Age	Injuries	Admission
72	Multiple brain contusions, radial head fracture, subarachnoid hemorrhage, subdural hematoma, comminuted nasal bone fracture, mandibular fracture	ICU
57	Subarachnoid hemorrhage	ICU
61	Elbow laceration, pneumothorax, rib fracture	
40	Comminuted intra-articular fracture of the tibial plateau with impaction, comminuted intra-articular fracture of the proximal fibula, partial tear of the Achilles tendon.	
62	Comminuted fracture of the proximal humerus, inferiorly displaced comminuted fracture of the right orbital floor, displaced comminuted fracture of the anterior medial and lateral maxillary sinus walls, subarachnoid hemorrhage	ICU
52	Closed head injury without loss of consciousness	ICU
25	Trimalleolar fracture	
45	Displaced fractures of the superior pubic ramus and inferior pubic ramus	
33	Non-displaced fracture of the anterior column of the left acetabulum, non-displaced fracture of the left inferior pubic ramus	
73	Mandibular fractures, comminuted and displaced fractures of the anterolateral and posterolateral walls of the left maxillary sinus, displaced fracture of the zygomatic arch, fracture of the left orbital floor, comminuted fracture of the lateral wall of the left orbit, angulated fracture of the left nasal bone, fracture of the lateral pterygoid plate.	
73	Comminuted transverse fracture of the left anterior column of the acetabular cup with femoral head displacement	
59	Femoral neck fracture	
58	Right pneumothorax, second, third and eighth rib fracture, right pulmonary contusion, right acetabular fracture, respiratory failure	ICU
55	Open distal fibula fracture	
57	Subarachnoid hemorrhage, Intraparenchymal contusion	ICU
55	Respiratory failure, right subdural hematoma, and basilar skull fracture	ICU

*ICU,* intensive care unit

**Table 2:** Compiled data from the National Electronic Injury Surveillance System on all Segway® injuries from 2009 through 2013. (n=33).

Age	Diagnosis
72	Tib/fib fracture
12	Fractured elbow
56	Right shoulder contusion
62	Left shoulder sprain
51	Intertrochanteric hip fracture
74	Abrasions
35	Humeral fracture
59	Fibular fracture
48	Wrist fracture
56	Pubic ramus fracture
12	Concussion
20	Abrasions
87	Nasal fracture
61	Radial head fracture, wrist fracture
45	Shoulder fracture
65	Leg hematoma
61	Closed head injury
13	Neck pain
86	Sprained knee
55	Sprained ankle
56	Fibular fracture
37	Lip laceration
59	Hand fracture
67	Wrist fracture
55	Elbow fracture
54	Hand laceration
22	Knee abrasions
43	Laceration
46	Back contusion
63	Ankle fracture
56	Facial laceration
86	Concussion
75	Abrasions

## References

[1] About Segway. Segway Inc. The Leader in Personal, Green Transportation.

[2] Centre for Electric Vehicle Experimentation in Quebec (2006). Pilot Project for Evaluating the Segway HT Motorized Personal Transportation Device in Real Conditions.

[3] Boniface K, McKay MP, Lucas R (2011). Serious injuries related to the Segway personal transporter: A case series. Ann Emerg Med.

[4] Vincent K, Block E, Black J (2009). Traumatic injuries associated with Segways and personal transporters. Am Surg.

[5] Mikkelsen R, Petersen A, Hvolris J (2014). Two cases of associated hip fractures after falls from the Segway Personal Transporter. Emerg Med.

[6] Barnes J, Webb M, Holland J (2013). The quickest way to A&E maybe via the Segway. BMJ Case Rep.

[7] National Electronic Injury Surveillance System (NEISS) http://www.cpsc.gov/en/Research--Statistics/NEISS-Injury-Data/.

[8] Williams S (2010). Segway Owner Dies in Segway Crash. Wheels Segway Owner Dies in Segway Crash Comments.

